# Draft Genome Sequence of a Strictly Anaerobic Dichloromethane-Degrading Bacterium

**DOI:** 10.1128/genomeA.00037-16

**Published:** 2016-03-03

**Authors:** Sara Kleindienst, Steven A. Higgins, Despina Tsementzi, Konstantinos T. Konstantinidis, E. Erin Mack, Frank E. Löffler

**Affiliations:** a University of Tennessee and Oak Ridge National Laboratory (UT-ORNL) Joint Institute for Biological Sciences (JIBS) and Biosciences Division, Oak Ridge National Laboratory, Oak Ridge, Tennessee, USA; b Center for Environmental Biotechnology, University of Tennessee, Knoxville, Tennessee, USA; c Department of Microbiology, University of Tennessee, Knoxville, Tennessee, USA; d Department of Civil and Environmental Engineering, University of Tennessee, Knoxville, Tennessee, USA; e School of Civil and Environmental Engineering, Georgia Institute of Technology, Atlanta, Georgia, USA; f School of Biology, Georgia Institute of Technology, Atlanta, Georgia, USA; g Corporate Remediation Group, E. I. DuPont de Nemours and Company, Newark, Delaware, USA

## Abstract

An anaerobic, dichloromethane-degrading bacterium affiliated with novel *Peptococcaceae* was maintained in a microbial consortium. The organism originated from pristine freshwater sediment collected from Rio Mameyes in Luquillo, Puerto Rico, in October 2009 (latitude 18°21′43.9″, longitude −65°46′8.4″). The draft genome sequence is 2.1 Mb and has a G+C content of 43.5%.

## GENOME ANNOUNCEMENT

Dichloromethane (DCM, methylene chloride) is an anthropogenic contaminant of groundwater that also occurs naturally, and is susceptible to aerobic and anaerobic degradation (1–5). DCM degradation under oxic and denitrifying conditions is well characterized (1, 6). In the absence of oxygen or nitrate, DCM degradation was associated with the formation of formate, acetate, and inorganic chloride (7–9). The only described pure culture capable of anaerobic DCM degradation is *Dehalobacterium formicoaceticum* (7). Enzyme assays using *Dehalobacterium formicoaceticum* extracts suggested that DCM metabolism proceeds via the Wood-Ljungdahl pathway; however, detailed mechanistic understanding and genome information is lacking (10).

A metagenome was generated from a microbial consortium, which utilized DCM as the sole energy source under anoxic conditions. DNA was extracted as described (11). A DNA library for Illumina MiSeq sequencing was constructed using the Nextera DNA sample preparation kit (Illumina, San Diego, CA, USA) and evaluated using the Qubit double-stranded DNA broad range reagent kit (Life Technologies, Foster City, CA, USA) with a Qubit fluorometer (Thermo, Fisher Scientific, Waltham, MA), a 7500 DNA kit on a 2100 Bioanalyzer instrument (Agilent Technologies, Santa Clara, CA, USA), and a KAPA SYBR FAST qPCR kit (KAPA Biosystems, Wilmington, MA, USA). Following manufacturer protocols, a 20-pM library was sequenced on an Illumina MiSeq sequencer using a v3 MiSeq reagent kit. The sequencing run produced 15,719,461 paired-end reads, which were filtered using the High-Throughput Quality Control (HTQC) toolkit (12) with a minimum phred score of 20 and a minimum read length of 100, yielding 13,003,937 paired-end reads. The quality-filtered reads were assembled with the Velvet (13), RAY (14) and Newbler v2.6 (Roche Applied Science, Penzberg, Germany) assemblers (15). Contigs longer than 500 bp (*n* = 32,063) were binned using MetaWatt (version 1.7) (16). Initial analyses of the contigs with MetaWatt identified a set of 42 contigs with distinct G+C content, tetranucleotide frequencies, and consistent association with publicly available genomes of the *Peptococcaceae* family (7.4% of all fragments). Reassembly of aligned metagenomic reads to the 42 binned contigs decreased contamination by 0.26% and resulted in a draft genome of 75 contigs with >500 nt. The obtained genome was evaluated for its completeness (92.47%) and contamination (4.44%) using CheckM v0.9.7, based on the detection of 420 single copy marker genes found in 35 *Clostridia* genomes (17). Analysis of tetranucleotide frequencies, coding density space, and G+C content distributions flagged 22 contigs as outliers, which were excluded from further analysis, and reduced the contamination estimate to 1.21%.

The final draft genome consisted of 53 contigs with total size 2,076,422 bp and a G+C content of 43.5%. Gene annotations were performed with the IMG pipeline (18), resulting in 2,395 predicted genes and 2,323 protein coding sequences. The genome encodes the Wood-Ljungdahl pathway (8 genes), hydrogenases (21 genes), sporulation (48 genes), and chemotaxis (11 genes) as well as motility proteins (i.e., a flagellum; 27 genes).

### Nucleotide sequence accession numbers.

The draft genome has been deposited at DDBJ/EMBL/GenBank under the accession number LNDB00000000. The version described in this paper is the first version, LNDB01000000.
